# Which Microbes Like My Diet and What Does It Mean for My Heart?

**DOI:** 10.3390/nu13114146

**Published:** 2021-11-19

**Authors:** Emilia Sawicka-Śmiarowska, Anna Moniuszko-Malinowska, Karol Adam Kamiński

**Affiliations:** 1 Department of Population Medicine and Lifestyle Diseases Prevention, Medical University of Bialystok, 15-269 Bialystok, Poland; medycyna.populacyjna@umb.edu.pl; 2 Department of Cardiology, Medical University of Bialystok, 15-276 Bialystok, Poland; 3 Department of Infectious Diseases and Neuroinfection, Medical University of Bialystok, 15-540 Bialystok, Poland; anna.moniuszko@umb.edu.pl

**Keywords:** gut microbiome, diet, heart, cardiovascular risk factors, cardiovascular diseases

## Abstract

Cardiovascular diseases are the most common causes of hospitalization, death and disability in Europe. Despite our knowledge of nonmodifiable and modifiable cardiovascular classical risk factors, the morbidity and mortality in this group of diseases remains high, leading to high social and economic costs. Therefore, it is necessary to explore new factors, such as the gut microbiome, that may play a role in many crucial pathological processes related to cardiovascular diseases. Diet is a potentially modifiable cardiovascular risk factor. Fats, proteins, carbohydrates, vitamins and minerals are nutrients that are essential to the proper function of the human body. The style and composition of the human diet has changed over time, evolving from a hunter–gatherer diet to an industrialized and Westernized modern diet that includes processed products. The relationship between the gut microbiome, diet and cardiovascular diseases is complex and still not fully understood. In this review, we discuss, in the context of diet, why particular microbes occur in individuals and how they can influence the host’s cardiovascular system in health and disease. We investigate the role of particular microorganisms and changes in the *Firmicutes/Bacteroidetes* ratio.

## 1. Introduction

Cardiovascular pathologies, among non-infectious diseases, are significant causes of mortality (49% of all deaths) [1]. Annually, 4 million deaths in Europe are attributed to these diseases and cardiovascular mortality is higher in Central and Eastern Europe compared with Western Europe [1]. Despite our knowledge of nonmodifiable and modifiable cardiovascular classical risk factors, reliably described in Framingham’s cohort study [2], the morbidity and mortality in this group of diseases remains high [3]. Due to the above-mentioned reasons, and the high social and economic costs related to this group of diseases, it is necessary to explore other factors, such as the gut microbiome, that may play a role in many crucial pathological processes in the cardiovascular system. The data from the literature revealed that bacteria form an interactive ecosystem of interdependencies with their host cells in the majority of cases, in the form of mutualism and commensalism [4,5,6]. This influences the immune processes of the host as well as metabolic homeostasis, and is therefore an important element in cardiovascular disease development and progression [7]. Diet is a cardiovascular risk factor that may be easily modifiable. The relationship between the gut microbiome, diet and cardiovascular disease is complex and still not fully understood.

The human microbiome is formed shortly after birth and in healthy individuals this process is largely completed within the first 3–5 years; however, it can be modified by diet [8,9]. Fats, proteins, carbohydrates and vitamins are nutrients that are essential to the proper functioning of the human body. The style and composition of the human diet has changed over time and evolved from a hunter–gatherers diet to an industrialized and Westernized modern diet including processed products and fast food. It is suggested that gut microbiota are involved in energy homeostasis by extracting calories from nutrients and in the metabolism of short-chain fatty acids, amino acids and vitamins [5].

In this review, we discuss the relationship between microbes and diet pattern components, and how their association can influence the host’s cardiovascular system. 

## 2. Diet pattern

### 2.1. Traditional vs. Modern Industrialized Diet

The industrialization of European countries has resulted in an increased amount of fat in our diet, and a reduction in complex carbohydrate consumption, which has led to a decrease in gut microbiome diversity [10]. It was found that African children from rural villages, in contrast to European populations, showed a significant enrichment in phylum *Bacteroidetes* and a depletion in *Firmicutes*, and the family of *Enterobacteriaceae* from the phylum *Proteobacteria*, with a unique abundance of bacteria from the genera *Prevotella* and *Xylanibacter*. The latter contains a set of bacterial genes for cellulose and xylan hydrolysis that are completely missing in European children [10]. Furthermore, their diet was rich in short-chain fatty acids [10]. Differences in the gut microbiome and metabolomics profile between unindustrialized, traditional rural lifestyles (Hadza hunter-gatherers of Tanzania) and the industrialized, Westernized diet and lifestyles of urban Italians were found [10,11,12,13]. The Hadza diet consists of wild foods that can be classified into five main categories: meat, honey, baobab, berries and tubers, without cultivated or domesticated plants and animals, and minimal amounts of agricultural products, whereas the diet of the Italian cohort is almost entirely composed of commercial agricultural products and adheres to the Mediterranean diet, which includes abundant plant foods, fresh fruit, pasta, bread and olive oil [11]. The study revealed higher abundance of *Bacteroidetes* and a lower abundance of *Firmicutes* in the Hadza population compared to the Italian group, and the near absence of *Actinobacteria*, an important subdominant component, in the Italian microbiome [11]. Furthermore, the Hadza gut microbial ecosystem was profoundly depleted in *Bifidobacterium*, enriched in *Prevotella*, and comprised of unusual arrangement of *Clostridiales* [11]. Moreover, a review by García-Montero et al. did not compare specific diets related to particular environmental factors but rather nutritional approaches such as the Mediterranean and Westernized diet [14]. Based on a PREDIMED (PREvención con DIeta MEDiterránea) study, the authors concluded that participants with better adherence to the Mediterranean diet, who consumed more polysaccharides and plant proteins and less animal protein [15] and those with reduced animal protein in their diet, presented a higher abundance of *Bacteroidetes* with a lower *Firmicutes/Bacteroidetes* ratio [14,15]. This diet pattern was related to greater diversity, and an improved gut barrier function and permeability [16]. Participants who consumed greater amounts of animal protein differed from the Mediterranean diet and were characterized by a higher *Firmicutes/Bacteroidetes* ratio [14,15].

The Westernized diet is characterized by a high content of unhealthy and harmful elements along with a reduced consumption of fruits and vegetables. This dietary approach promotes a pathological microbiota status, leading to a higher *Firmicutes/Bacteroidetes* ratio [14].

#### What Does It Mean for the Heart?

**Figure 1:**
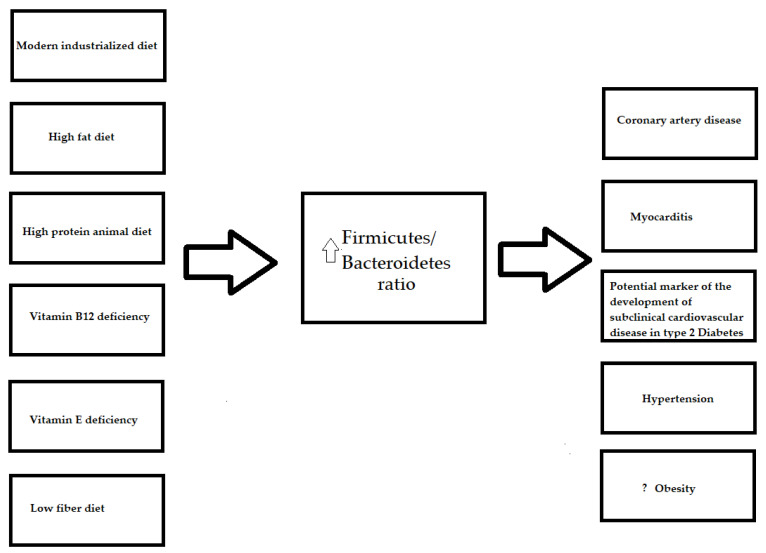
The influence of the diet on *Firmicutes/Bacteroidetes* ratio and diseases. ?—the relationship not fully elucidated; uparrow (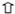)—an increase.

The gut microbiota of adults mainly consists of *Firmicutes* and *Bacteroidetes* that, together with *Actinobacteria* and *Proteobacteria,* account for nearly 99% of the intestinal microbiome [17]. It is worth noting that diet is suggested to be related to changes in all four of the main gut microbiome phyla (Table 1), although the authors paid particular attention to the abundance of *Firmicutes* and *Bacteroidetes*. The *Firmicutes/Bacteroidetes* ratio is considered to be an indicator for gut dysbiosis. A higher ratio was found for an industrialized diet (Figure 1) [18], and was found to be associated with coronary artery disease [12,13] and myocarditis [19]. Furthermore, it was proven that fecal microbiota transplantation, a method that is used to restore gut homeostasis, alleviates myocardial damage in myocarditis [19].

## 3. Diet Compound

### 3.1. Fats

The relationships between dietary fat and health are complex. On the one hand, diets high in saturated and trans fatty acids increase the risk of cardiovascular diseases via the up-regulation of total blood cholesterol and LDL-cholesterol [20]. On the other hand, mono- and polyunsaturated fats are beneficial to our health.

The contribution of the gut microbiome to fat metabolism is multidirectional. Gut microbiota (such as *Eubacterium* and *Bacteroides*) are proven to participate in the conversion of cholesterol to coprostanol, which is a non-absorbable sterol excreted in the feces. Two major pathways have been proposed for this process. The first involves a direct reduction in the 5–6 double bond in cholesterol, while the second consists of the oxidation of the 3β-hydroxy group and the isomerization of the double bond to yield 4-cholesten-3-one, which undergoes two reductions to form coprostanone and then coprostanol [21]. Furthermore, diets rich in fats stimulate the production of bile acids that, in addition to their role in the absorption of dietary lipids and lipid-soluble nutrients, exhibit antimicrobial activity and promote microbiome species capable of metabolizing bile acids in the intestine [21]. Microbiota also participates in the transformation of primary bile acid to secondary bile acid via the deconjugation, oxidation and epimerization of hydroxyl groups at C3, C7 and C12, 7-dehydroxylation, esterification and desulfatation [21]. Different secondary bile acids can influence health in various ways [21].

The human diet is important for the gut microbiome composition in both adult life and during childhood and even before birth. A study by Chu et al. revealed that, independent of maternal body mass index, a maternal high-fat diet is associated with distinct changes in the neonatal gut microbiome at birth, which persists through 4–6 weeks of age [22]. The study showed that a diet rich in fat caused a reduction in the *Bacteroides* species in the infant gut [22]. These microbes are responsible for the polysaccharide metabolism, including for the production of short-chain fatty acids from human milk oligosaccharides, which is a major energy source for rapidly growing infants [23] and affects their immune development [23]. Higher dietary fat content also resulted in an increase in *Firmicutes, Proteobacteria* and *Actinobacteria* in mice [24,25,26,27]. The data concerning *Bacteroidetes* in the context of a fat rich diet are ambiguous [24,26,28]. This might be related to the fact that, as shown by Wu et al., among *Bacteroidetes*, *Bacteroides* species are associated with long-term proteins and animal-fat consumption, whereas increased levels of *Prevotella* were found in patients with diets high in carbohydrates, especially fiber [29]. Furthermore, it was proven that a high-fat diet increased the abundance of Gram-negative bacteria [30].

Additionally, a meta-analysis based on 27 dietary studies, including 1,101 samples from rodents and humans in a finer taxonomic analysis, revealed that the most reproducible signals of a high-fat diet are *Lactococcus* species (*Firmicutes*), which were demonstrated to be common dietary contaminants [31]. Furthermore, in this study, the *Firmicutes/Bacteroidetes* ratio was significantly correlated with fat content [31] (Figure 1).

#### What Does It Mean for the Heart?

Gut microbiome changes, related to high-fat diets, are presented in Table 2. An increase in *Lactococcus,* which belong to *Firmicutes* phylum and participate in glucose fermentation and, therefore, lactic acid production, was shown to have both pathogenic and protective capabilities. It was demonstrated that milk fermented by *Lactococcus lactis* imparts an important systolic and diastolic blood pressure and HR-lowering effect [32]. 

A high fat diet is suspected to play a role in chronic low-grade inflammation, which can cause atherosclerosis in many cardiovascular diseases. It has also been suggested to be related to an increased abundance of Gram-negative bacteria, which contain lipopolysaccharides on their outer membrane (LPS) [53]. The lipid A component of LPS binds to Toll-like receptor 4 (TLR-4) [53], leading to the activation of nuclear factor kappa B (NF-κB) signaling the release of pro-inflammatory cytokines [54]. TLR-4 may also be stimulated directly by free fatty acids [55]. In addition, high-fat diets increase barrier-disrupting cytokines (tumor necrosis factor alpha (TNF α), interleukin (IL) 1B, IL6, and interferon γ) and decrease barrier-forming cytokines (IL10, IL17, and IL22) [56]. These mechanisms increase gut permeability and promote LPS, free fatty acids and the infiltration of pro-inflammatory cytokines into the circulation [57]. 

What is more, in a mice study, changes of the gut microbiota induced by a long-term high-fat diet were associated with increased intestinal oxidative stress expressed as reactive oxygen species, total antioxidant capacity, and malondialdehyde [58].

**Figure 2:**
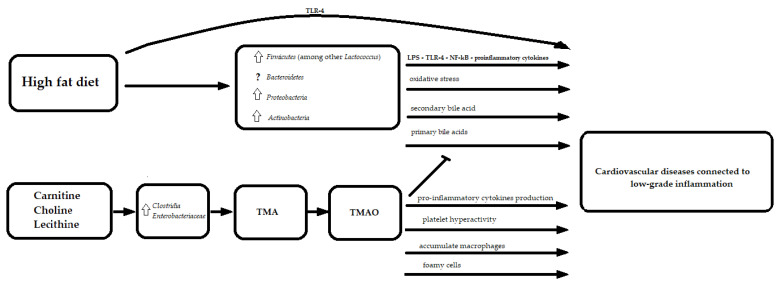
The molecular mechanisms linking fat in diet, gut microbiota and cardiovascular diseases. TMA—trimethylamine; TMAO—trimethylamine N-oxide; LPS—lipopolysaccharide; TLR-4—Toll-like receptor 4; NF-kB—Nuclear Factor kappa-light-chain-enhancer of activated B cells; ?—the relationship not fully elucidated; uparrow (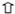)—an increase.

Furthermore, the previously mentioned bile acids are recognized as signaling molecules through the activation of receptors such as the farnesoid X receptor (FXR) or G protein-coupled receptor (TGR5) [59]. Moreover, the inhibition of bile acid synthesis in the liver might be regulated by the gut microbiota-dependent expression of fibroblast growth factor 15 in the ileum and cholesterol 7α-hydroxylase (CYP7A1) in the liver by FXR-dependent mechanisms [60]. In the literature, the tauro-conjugated beta- and alpha-muricholic acids were identified as FXR antagonists [60]. The activation of vasculature-specific FXR improves lipid profiles and influences vascular tension, thereby resulting in an anti-atherosclerotic effect [61]. On the other hand, FXR expression was significantly up-regulated in the ischemic cardiac tissue in rats, and the inhibition of FXR reduced the size of the insult, suggesting that FXR plays an important role in mediating cardiac apoptosis and injury [61] (Figure 2).

Furthermore, it was shown that trimethylamine N-oxide (TMAO) induces atherosclerosis by reducing cholesterol metabolism through hepatic bile acid inhibition [62].

TMAO is a product of trimethylamine (TMA) oxidation via hepatic flavin monooxygenases. TMA is produced by gut microbes, such as *Clostridia* and *Enterobacteriaceae*, from carnitine, choline, and lecithin, which can be found in various dietary products including red meat and eggs [63]. The proatherosclerosis function of TMAO might be related to two macrophage scavenger receptors (cluster of differentiation 36 and scavenger receptor A) leading to the accumulation of macrophages, foamy cells formation and the promotion of inflammation [64,65,66,67]. The CD36/MAPK/JNK pathway may play a crucial role in the formation of TMAO-induced foam cells [68]. TMAO can cause increased pro-inflammatory cytokines production, such as TNF α and IL-1B, while decreasing anti-inflammatory cytokines such as IL-10 [69]. Moreover, TMAO was reported to induce platelet hyperactivity, which could lead to atherosclerotic thrombotic events [70]. Therefore, TMAO is considered to be one of pivotal mechanisms through which the diet and microbiome may affect the development of atherosclerosis (Figure 2).

### 3.2. Proteins

Dietary proteins are considered to be one of the most important macronutrients. They are digested to produce amino acids, which are used for protein synthesis. They have many important functions in the human body, including in the cardiovascular system, for instance, for transport (hemoglobin) and muscle contraction (actin, myosin). The undigested proteins and amino acids are mainly fermented by bacteria into various metabolites, such as organic amines, hydrogen sulfate and ammonia, both of which participate in various physiological functions related to host health and diseases. Greater levels of undigested proteins lead to an increase in pathogenic microorganism with an associated higher risk of metabolic diseases [71]. Both the origin of the protein (animal vs. plant) and its concentration can influence the gut microbiome [71]. It was shown that a diet enriched with 20% peanut protein led to an increase in *Bifidobacterium* and a reduction in *Enterobacteria* and *Clostridium perfringensa* in rats [33]. Furthermore, a higher intake of soybean protein was associated with an increase in *Escherichia* and *Propionibacterium* abundance [34,35,36], whereas data from the literature revealed that casein, a protein present in milk, can also increase the abundance of *Bifidobacterium* and *Lactobacilli* [37], and decrease the abundance of *Staphylococci, Coliformis, Streptococci* [38], *Eubacterium rectale* and *Marvinbryantia formatexigens* [39]. Moreover, in the study of Garcia-Mantrana I. et al. a higher intake of animal protein was found to be related to a significantly lower presence of *Bacteroidetes* (*p* = 0.029) and a higher *Firmicutes/Bacteroidetes* ratio [15].

Moreover, a randomized, double-blind, parallel-design trial conducted in 38 overweight individuals, who received high-protein isocaloric diets with casein, soy protein or a maltodextrin (control) supplementation, revealed that high-protein diets did not alter the microbiota composition, but induced a shift in bacterial metabolism toward amino acid degradation with different metabolite profiles in accordance with the protein source [72]. Another study suggests that the dietary protein/fat ratio has only minimal, transient effects on the gut microbiota composition; however, gut microbiota might contribute to variability in host responses to high altitude [73]. Moreover, a positive correlation was found between *Firmicutes* abundance and high concentrations of amino acid degradation products, that might be related to *Clostridia* activity [74] and a negative correlation was identified for an abundance of *Bacteroidetes* with the exception for *Butyricimonas* and *Odoribacter* genera, both of which showed a positive correlation [72]. A low amount of protein in the diet, by reducing substrate for pathogenic bacteria proliferation, resulted in reduced *Escherichia coli* abundance on the mucosal surface [75,76,77]. The gut microbiome composition in animal models seems to be strictly associated with the consumption of dietary protein, whereby doses from 100 to 200 g/kg caused an increase in *Lactobacilli* and a reduction in *Coliformis* and *Staphylococci*; doses greater than 200 g/kg caused an increase in *Coliforms, Streptococcus* and *Bacillus* [40].

The above-mentioned studies confirm proteins’ role in regulating the gut microbiome; however, the study based on 109 healthy men and women aged 21 to 65, with a body mass index of 18 to 36, revealed that protein is less influential than saturated fat [78].

#### What Does It Mean for the Heart?

Both the type and the amount of proteins in the diet seem to play a role in gut microbiome composition, which influences our health and, more specifically, the heart (Table 2). 

Diets rich with plants as well as animal protein were associated with an increase of some genus of *Firmucutes* and *Actinobacteria*, particularly with a high abundance of *Bifidobacterium,* which, together with *Lactobacilli,* are used as probiotics. Increased *Bifidobacterium* contributes to the generation of more microbial metabolites, including acetic acid and lactic acid, resulting in a lower pH in the gut, that inhibits toxic metabolite products, such as amine and benzpyrole. Its increased abundance was shown in heart failure patients [79], while its protective role in myocardium infarction was proven in mice models [80]. Pretreatment with *Bifidobacterium animalis subsp. lactis 420* for 10 or 28 days attenuated cardiac injury from ischemia-reperfusion and reduced levels of inflammatory markers, which was mediated by Treg immune cells in mice models [80]. Furthermore, the symbiotic supplementation of *Lactobacillus acidophilus*, *Lactobacillus casei*, and *Bifidobacterium bifidum* for 12 weeks among people who are overweight, or have diabetes and coronary heart disease had beneficial effects on serum hs-CRP, plasma nitric oxide, and plasma malondialdehyde levels [81]. The supplementation did not have any effect on other biomarkers of oxidative stress levels and on carotid intima-media thickness [81]. Unfortunately, in this study, gut microbiome composition was not investigated. The oral supplementation of *Lactobacillus rhamnosus* significantly improved outcomes following ischemia-induced heart failure in rats [82] and attenuated the induction of hypertrophy in cardiomyocytes [83]. 

In an animal model, a dietary intake of protein of up to 200 g/kg was related to changes in the gut microbiome through a reduction of pathogenic bacteria *Coliformis (Citrobacter, Enterobacter, Hafnia, Klebsiella, Escherichia*) and *Staphylococci,* whereas an increase in protein intake was related to an increase in the abundance of those bacteria [61]. These Gram-negative bacteria affect homeostasis through LPS production. Furthermore, some of these bacteria (*Klebsiella* and *Streptococcus*) were found in atherosclerotic lesions [84]. In mice models, three secreted products, namely, staphylococci–coagulase, the von Willebrand factor binding protein and the clumping factor were proven to promote thromboembolic lesions in heart tissues in sepsis [85]. The first two are known to activate prothrombin to cleave fibrinogen, while clumping factor allowed *Staphylococci* to associate with the resulting fibrin [85].

**Figure 3:**
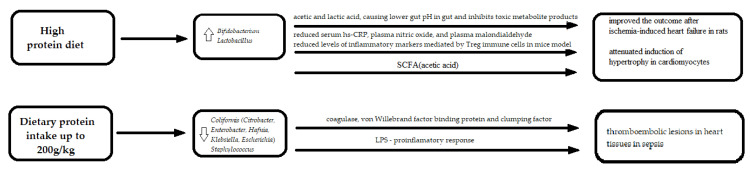
The molecular mechanisms linking protein in diet, gut microbiota and cardiovascular diseases. LPS—lipopolysaccharide; SCFA—Short-Chain Fatty Acid; CRP—c-reactive protein; uparrow (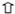)—an increase; downarrow (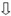)—a decrease.

Interestingly, high concentrations of the amino acid degradation products were correlated directly with two genera from the *Odoribacteraceae* family, which seem to have different relationships with blood pressure [72]. On the one hand, they were positively related to *Butyricimonas,* which was shown to have a positive correlation with mean arterial pressure in elderly women from South Korea [86]. On the other hand, *Odoribacter,* a short-chain fatty acid-producing bacteria, was proven to help maintain lower systolic blood pressure in pregnant women through butyrate production [87], and in rats [88]. Despite the positive role of *Odoribacter* in controlling blood pressure, it was directly correlated with aortic aneurysm diameters in mice [89] (Figure 3).

### 3.3. Carbohydrates

Daily energy requirements depend on gender, age, height, weight, health, and levels of physical activity. In both the prevention and treatment of cardiovascular diseases, maintaining a particular body weight is extremely important. Carbohydrates, as the main source of energy in the diet, are crucial for energy homeostasis. 

Feva et al. studied 88 subjects at an increased risk of metabolic syndrome, who were kept on a high saturated fat diet for 4 weeks (baseline), and were then randomly placed under one of the five experimental diets for 24 weeks [41]. The study revealed that low fat, high carbohydrate diets increased fecal *Bifidobacterium* and high carbohydrate/high glycemic index increased fecal *Bacteroides*, whereas high carbohydrate/low glycemic index and a high saturated diet increased *Faecalibacterium prausnitzii*. Changes in the abundance of fecal *Bacteroides* correlated inversely with body weight [41]. Another study showed that supplementing the diet with galacto-oligosaccharides increased the abundance of *Bifidobacterium* species in feces five-fold in obese or overweight people [42].

Furthermore, an analysis conducted on 1135 participants from a Dutch population, using deep sequencing [43], showed that a higher intake of total carbohydrates were most strongly associated with an increase in *Bifidobacteria,* while they were associated with a decrease in *Lactobacillus, Streptococcus*, and *Roseburia* [43]. *Lactobacillus* and *Streptococcus* are lactose-fermenting bacteria that, through lactic acid production, lower the gut pH and inhibits amine and benzopyrole production, while *Roseburia* lead to increasing serum and fecal butyrate levels via the conversion of acetate into butyrate [90].

#### 3.3.1. Dietary Fiber

Fiber contains a mixture of polysaccharide and non-polysaccharide substances (such as lignins, cutins) that cannot be digested by enzymes of the human digestive tract. Instead, it is metabolized by microbes that generate short-chain fatty acids (SCFAs), including acetate, propionate and butyrate. Most of the produced acetate and propionate is absorbed by the gut, while butyrate is used by colon mucosa cells as a primary energy source [91].

The effects of fiber in the gut microbiome were summarized in a systematic review and meta-analysis from 2018, which was based on a total of 64 studies involving 2099 participants [44]. Dietary fiber intervention resulted in a higher abundance of *Bifidobacterium* spp., *Lactobacillus* [44,47] and the fecal butyrate concentration [44]. Moreover, a subgroup analysis revealed that fructans and galactooligosaccharides also led to a significantly greater abundance of the same bacteria [47]. Another study based on whole-genome shotgun sequencing revealed that fiber consumption shifted the *Bacteroidetes/Firmicutes* ratio, increasing the relative abundance of *Bacteroidetes* [62]. Furthermore, an analysis of correlations showed a positive relation between the *Bacteroidetes/Firmicutes* ratio and total dietary fiber intake but not with body mass index [47].

Notably, in mice studies, diets with navy bean and black bean flours rich in non-digestible fermentable carbohydrates increased the abundance of carbohydrate fermenting bacteria such as *Prevotella, S24-7* and *Ruminococcus flavefaciens*, which coincided with enhanced short-chain fatty acid production and colonic expression of the SCFA receptors GPR-41/-43/-109a [46]. 

#### 3.3.2. What Does It Mean for the Heart?

Similarly to a high-protein diet, high-carbohydrate and high-fiber diets resulted in an increase in the genus *Bifidobacterium* from phylum *Actinobacteria*, that imparts positive effects on health and the heart was previously discussed. Furthermore, a high-carbohydrate and high-fiber diet was also related to an increase in *Bacteroidetes*, which caused a decrease in *Firmicutes/Bacteroidetes* ratio. As mentioned previously, a higher *Firmicutes/Bacteroidetes* ratio was related to an industrialized diet and many cardiovascular diseases [12,13,19]. A particular genus, *Prevotella,* from the phylum *Bacteroidetes*, associated with a diet rich in non-digestible carbohydrates, seems to also have a negative influence on health despite its positive value. It also plays an important role in dysbiosis in pre- and hypertension patients [92]. This genus was increase in high lifetime cardiovascular disease risk profile Bogalusa Heart Study participants [93] and *Prevotella copri* was proven to be associated with cardiac valve calcification [94]. 

Furthermore, after a high-fiber diet, a higher abundance of SCFAs intestinal producers was revealed. SCFAs (acetate, propionate and butyrate) are generally considered as favorable to health and their role was described in cardiovascular pathologies, including in hypertension [95,96] and atherosclerosis [95]. Interestingly, despite the fact that SCFA-producing bacteria were associated with lower blood pressure [97,98], the SCFAs concentration of stool was associated with higher blood pressure [97,98,99]. In order to elucidate this issue, studies concerning both the fecal and serum measurement of SCFAs should be included in future research.

Animal models suggest that SCFAs act via fatty acid receptors FFAR2 (GPR43), FFAR3 (GPR41) [100], olfactory receptor 78 (the human analogue-OR51E2) [101] and GPR109A (hydroxycarboxylic acid receptor (HCA) 2) [102]. The expression of these receptors differs between tissues and cell types, and it is present in intestinal epithelial cells, immune cells, and adipocytes. Propionate was proven to be the most potent agonist for both FFAR3 and FFAR2. Acetate was more selective for FFAR2, whereas butyrate and isobutyrate were more active on FFAR3 [100]. FFAR2 expressed in the renal arteries causes vasodilatation in response to SCFAs, whereas Olfr78 mediated higher blood pressure through rennin release from granules in the renal juxtaglomerular apparatus [103]. The potency of SCFAs is much lower for Olfr78 and OR51E2, than for FFAR2, and therefore, it was suggested that Olfr78 serves as a negative feedback loop for the blood pressure, reducing the effects of FFAR2 [101]. Furthermore, propionate, in the absence of one of two SCFAs sensory receptors (Olfr78 and FFAR3), differentially modulated blood pressure [104]. SCFAs have also been suggested to be implicated in gut–brain communication, via the expression of butyrate receptors in the hypothalamus [105] and vagal afferents express receptors [106].

Furthermore, a study of mice models with transcriptome analyses showed that the protective effects of a high-fiber diet and acetate supplementation were accompanied by the down-regulation of cardiac and renal Egr1, which is involved in cardiac hypertrophy, cardio renal fibrosis, and inflammation [107]. Furthermore, a recently published article confirms fiber’s role in blood pressure reduction that it is associated with sympatholytic negative inotropic effects, in vivo [108]. 

Propionate supplementation in mice models resulted in attenuated cardiac hypertrophy, fibrosis, vascular dysfunction and hypertension [109]. This effect was related to reduced systemic inflammation, which is defined as a reduction in splenic effector memory T cell frequencies and splenic T helper 17 cells [109]. 

**Figure 4:**
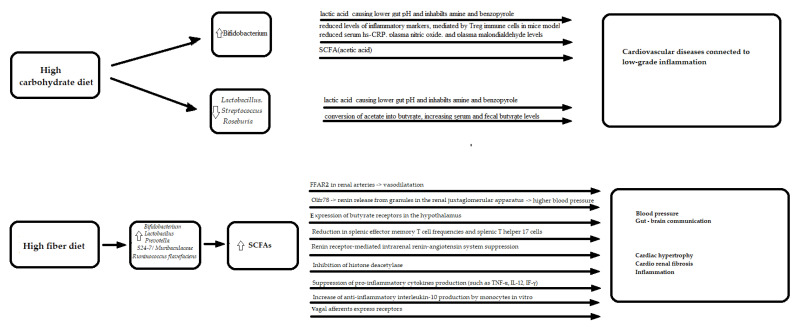
The molecular mechanisms linking carbohydrate in diet, gut microbiota and cardiovascular diseases. LPS—lipopolysaccharide; SCFA—Short-Chain Fatty Acid; CRP—c-reactive protein; FFAR2—Free fatty acid receptor 2; Olfr 78—olfactory receptor 78; TNF—Tumor necrosis factor, IL—interleukin; uparrow (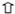)—an increase; downarrow (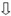)—a decrease.

The antihypertensive effect of sodium butyrate supplementation in rat models was related to (pro) renin receptor-mediated intrarenal renin-angiotensin system suppression [110] as well as anti-inflammatory effects that are presumed to be mediated by the inhibition of histone deacetylase [110]. Butyrate suppresses the production of pro-inflammatory cytokines, such as tumor-necrosis factor-α (TNF-α), interleukin-12 (IL-12), and interferon-γ (IF-γ), and up-regulates the production of anti-inflammatory interleukin-10 (IL-10) by monocytes, in vitro [110] (Figure 4).

### 3.4. Vitamins

Data concerning vitamins, the gut microbiome and cardiovascular diseases combined are limited in the literature. Among vitamins, vitamin D is the most widely described in the literature in this context. Although vitamin D has been traditionally recognized as a vitamin responsible for bone–mineral health, its role in hypertension, peripheral artery disease, myocardial infarction and heart failure has also been described [111].

In a cohort of 34 hypertension patients and 15 healthy controls, Zuo et al. suggested that gut microbiome dysbiosis, contributing to the hypertension development, might be partially mediated by vitamin D3 deficiency [112]. Vitamin D3 was significantly decreased in the feces of hypertension patients and correlated with decreased biodiversity, expressed through the Shannon index and Pielou evenness [112]. Moreover, fecal vitamin D3 positively correlated with *Subdoligranulum*, *Ruminiclostridium*, *Intestinimonas*, *Pseudoflavonifractor*, *Paenibacillus*, and *Marvinbryantia* genera from *Firmicutes* [112]. A recently published article on vitamin D and gut the microbiome includes a study of 567 older men, and identified eight taxa in the *Firmicutes* phylum that were positively associated with vitamin D metabolites. In the study, men with higher levels of calcitriol and higher calcitriol activation ratios, but not calcifediol, were more likely to possess butyrate-producing bacteria that are associated with better gut microbial health [48] and clinical status.

A recent human study on twenty adults with vitamin D insufficiency or deficiency, who were given 600, 4000 or 10,000 IU/day of oral vitamin D3, revealed that a higher baseline vitamin D concentration was associated with an increased relative abundance of *Akkermansia* (phylum *Verrucomicrobia*) and a decreased relative abundance of *Porphyromonas* (phylum *Bacteroidetes*), whereas after the intervention, dose-dependent increases in the relative abundance of *Bacteroides* (phylum *Bacteroidetes*) and *Parabacteroides* (phylum *Bacteroidetes*) were found [113].

**Figure 5:**
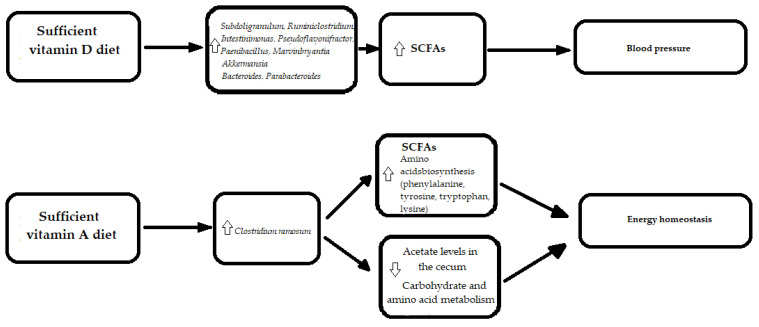
The molecular mechanisms linking vitamins in diet, gut microbiota and cardiovascular diseases. SCFAs—Short-Chain Fatty Acids. uparrow (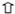)—an increase; downarrow (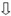)—a decrease.

For vitamin A, a relationship with formation of congenital heart defects [49], and energy homeostasis [114] were revealed. A study concerning vitamin A and the gut microbiome in mice models showed that mice with a vitamin A-deficient diet had significantly lower butyrate levels and higher acetate levels in the cecum compared to mice with a vitamin A-sufficient diet. The increase in butyrate levels in mice with a vitamin A-sufficient diet corresponded to higher numbers of the butyrate-producing bacteria—*Clostridium ramosum* (a member of *Clostridium_XVIII*), than for mice with a vitamin A-deficient diet. Mice with a vitamin A-deficient diet demonstrated disturbances in multiple metabolic pathways, including alterations in energy homeostasis [114]. Furthermore, a PICRUSt analysis revealed that bacteria from vitamin A-sufficient mice resulted in the enrichment of genes that are important in the biosynthesis of some amino acids, including phenylalanine, tyrosine, tryptophan, and lysine, while bacterial communities from vitamin A-insufficient mice had an enhanced carbohydrate and amino acid metabolism [114] (Figure 5).

Moreover, studies on vitamin B12 indicated that supplementation with cobalamin and whey increased *Bacteroidetes/Firmicutes* ratio and reduced *Proteobacteria* spp. abundance (*Escherichia/Shigella* spp. and *Pseudomonas*) [50]. In the study, cobalamin was found to enhance the growth of *Bacteroidetes*; meanwhile whey protein promoted the growth of *Firmicutes* [50].

In mice models, the low-vitamin E group was characterized with higher *Firmicutes/Bacteroidetes* ratio [51]. Furthermore, contrary to the control group, in the low- and high-vitamin E groups, *Escherichia coli* was found to be present [51].

#### What Does It Mean for the Heart?

The most widely described association in the literature is between fat-soluble vitamins (A, D, E, and K) and the gut microbiome. The above-mentioned data suggest that a higher concentration of vitamin D resulted in an increase in genera from *Firmicutes* phylum, which are responsible for lactic acid and butyrate production and are, therefore, supportive to our health. Furthermore, vitamin D concentrations were positively associated with an increased relative abundance of *Akkermansia* (phylum *Verrucomicrobia*), which was proven to be a beneficial microbe in terms of obesity, glucose and lipid metabolism [52]. In humans with a high body weight, blood cholesterol and fasting blood glucose level, the abundance of *Akkermansia* in the gut was lower than in healthy individuals [115]. Moreover, in mouse models, the high level of blood lipopolysaccharide, an indicator of intestinal permeability, decreased with the administration of *Akkermansia* [116].

Vitamin E and B12 deficiencies, lead to an increased *Firmicutes/Bacteroidetes* ratio, which has been described in many cardiovascular diseases (Figure 1).

### 3.5. Firmicutes/Bacteroidetes Ratio and Diet

Despite findings, with regard to the profiles of particular microorganisms in relation to diet, differing between studies, the most common result is the change in the *Firmicutes/Bacteroidetes* ratio. The *Firmicutes/Bacteroidetes* ratio, which is considered to be an indicator for gut dysbiosis, seems to increase in an unhealthy diet pattern, such as the modern industrialized diet, which includes a high amount of fat, animal protein and a lack of vitamin B12 and E (Figure 1). A higher *Firmicutes/Bacteroidetes* ratio is related to many inflammatory and metabolic disorders, including cardiovascular pathologies [12,13,19,117,118,119,120,121]. Although, in general, results of the intervention and observational studies confirm *Bacteroidetes* decrease and *Firmicutes* increase in obese humans [122] and animals [123], a meta-analysis performed by Sze M.C. et al. did not confirm such an association [124]. Therefore, further interventional studies across a large population are required to elucidate the relationship between the *Firmicutes/Bacteroidetes* ratio and obesity.

## 4. Summary 

In this review, we discussed the occurrence of microbes in individuals in relation to diet and how such microbes influence the host’s cardiovascular system in health and disease. 

We indicated that many bacteria are connected to diet patterns as well as to particular diet compounds. Furthermore, we related an unhealthy diet to an increased *Firmicutes/Bacteroidetes* ratio, which is considered an indicator for gut dysbiosis. Nevertheless, it is still not fully understood, as of yet, whether this pattern of dysbiosis is a cause or an effect of an improper diet.

Despite the amount of knowledge about diet as a modifiable cardiovascular risk factor and of the gut microbiome, in terms of the cardiovascular system, some influences, interdependencies and relationships are still not fully elucidated. Therefore, studies based on a larger population with advanced, novel “omics’ methods should be conducted.

## 5. Future Area of Interest and Goals

It is extremely important to remember that the gut microbiome composition is susceptible to many environmental factors other than diet, for instance, medication, tobacco smoking, age and gender. Therefore, all the above-mentioned patterns of dependencies and relationships should be verified in replication studies in different populations and environments. Nonetheless, it is crucial to underline that, in the future, particular attention should be paid to interventional studies based on diet modulation and microbiota transplantation. Another challenge is presented by the role played by genetic and environmental factors (temperature, habits as well as treatment and comorbidities) in the diet–microbiome–host relationship.

Furthermore, many molecular mechanisms concerning diet, gut microbiota and cardiovascular diseases have only been tested in animal models. The significance of these phenomena should be explored in humans with an emphasis on the distinctiveness of the gut microbiota in individual species.

## Figures and Tables

**Table 1:** The relation between diet pattern and gut microbiome composition.

Pattern of the Diet	Increase in Microbiome	Decrease in Microbiome
Traditional diet	- phylum *Bacteroidetes* (including genus *Prevotella* and *Xylanibacter)* [10,11] - unusual arrangement of *Clostridiales* [11]	- *Firmicutes/Bacteroidetes* ratio [14,15] - phylum *Firmicutes* [11] - family *Enterobacteriaceae* (phylum Proteobacteria) [10] - phylum *Actinobacteria* (including genus *Bifidobacterium*) [11] - unusual arrangement of *Clostridiales* [11]
Modern industrialized diet	- *Firmicutes/Bacteroidetes* ratio [14,15]	- gut microbiome diversity [10]

**Table 2:** The relation between diet compound and gut microbiome composition.

Pattern of the Diet	Increase in Microbiome	Decrease in Microbiome
High fat diet	- *Firmicutes/Bacteroidetes* ratio [31] - *Bacteroides* (phylum *Bacteroidetes*) - in adults [29] - *Lactococcus species* (phylum *Firmicutes*) [31] - phylum *Firmicutes*, *Proteobacteria*, *Actinobacteria* in mice [24,25,26,27]	- genus *Bacteroides* (phylum *Bacteroidetes*) in infants [22]
High protein plant diet	- genus *Bifidobacterium* (phylum *Actinobacteria*) in rats [33] - genus *Escherichia* (phylum *Proteobacteria*) [34,35,36] - genus *Propionibacterium* (phylum *Actinobacteria)* [34,35,36]	- family *Enterobacteriaceae* (phylum *Proteobacteria*), species *Clostridium perfringensa* (phylum *Firmicutes*) in rats [33]
High protein animal diet	- *Firmicutes/Bacteroidetes* ratio [15] - genus *Bifidobacterium* (phylum *Actinobacteria*) [37] - genus *Lactobacilus* (phylum *Firmicutes*) [37]	- genus *Bacteroidetes* (phylum *Bacteroidetes*) [15] - genus *Staphylococcus* (phylum *Firmicutes*) [38] - *Coliforms* (phylum *Proteobacteria*) [38] - genus *Streptococcus* (phylum *Firmicutes*) [38] - species *Eubacterium rectale* (phylum *Firmicutes*) [39] - strain *Marvinbryantia formatexigens* (phylum *Firmicutes*) [39]
Dietary protein amount: 100 to 200 g/kg	- genus *Lactobacillus* (phylum *Firmicutes*) [40]	- *Coliformis* (*Citrobacter, Enterobacter, Hafni, Klebsiella, Escherichia*) [40] - genus *Staphylococcus* (phylum *Firmicutes*) [40]
Dietary protein amount: dose greater than 200 g/kg	- genus *Bacillus* (phylum *Firmicutes*) [40] - genus *Streptococcus* (phylum *Firmicutes*) [40] - *Coliformis* (*Citrobacter*, *Enterobacter*, *Hafni*, *Klebsiella*, *Escherichia*) [40]	
High carbohydrates diet	- genus *Bifidobacterium* (phylum *Actinobacteria)* [41,42,43]	- genus *Lactobacilus* (phylum *Firmicutes*) [43] - genus *Streptococcus* (phylum *Firmicutes*) [43] - genus *Roseburia* (phylum *Firmicutes*) [43]
High fiber diet	- genus *Bifidobacterium* (phylum *Actinobacteria*) [44,45] - genus *Lactobacilus* (phylum *Firmicutes*) [44,45] - genus *Prevotella* (phylum *Bacteroidetes*) [46] - *S24-7*/*Muribaculaceae* (phylum *Bacteroidetes*) [46] - species *Ruminococcus flavefaciens* (phylum *Firmicutes*) [46]	- *Firmicutes/Bacteroidetes* ratio [47]
Diet sufficient with vitamin D	- gut microbiome diversity - *Subdoligranulum, Ruminiclostridium, Intestinimonas, Pseudoflavonifractor, Paenibacillus, Marvinbryantia* (phylum *Firmicutes*) [48] - genus *Akkermansia* (phylum *Verrucomicrobia*) [49] - genus *Bacteroides, Parabacteroides* (phylum *Bacteroidetes*) [49]	- genus *Porphyromonas* (phylum *Bacteroidetes*) [49]
Diet sufficient with vitamin A	- genus *Clostridium ramosum* (phylum *Firmicutes*) [50]	
Diet sufficient with vitamin B12	- phylum *Bacteroidetes* [51]	- *Firmicutes/Bacteroidetes* ratio [51] - genus *Escherichia/Shigella* spp. (phylum *Proteobacteria*) [51] - genus *Pseudomonas* (phylum *Proteobacteria*) [51]
Diet sufficient with vitamin E		- *Firmicutes/Bacteroidetes* ratio [52]

## Data Availability

Not applicable.
